# A long-term retrospective study on rehabilitation of seabirds in Gran Canaria Island, Spain (2003-2013)

**DOI:** 10.1371/journal.pone.0177366

**Published:** 2017-05-05

**Authors:** Natalia Montesdeoca, Pascual Calabuig, Juan A. Corbera, Jorge Orós

**Affiliations:** 1 Department of Animal Pathology, Veterinary Faculty, University of Las Palmas de Gran Canaria, Arucas, Las Palmas, Spain; 2 Tafira Wildlife Rehabilitation Center, Cabildo de Gran Canaria, Tafira Baja, Las Palmas de Gran Canaria, Spain; 3 Department of Morphology, Veterinary Faculty, University of Las Palmas de Gran Canaria, Arucas, Las Palmas, Spain; International Bird Rescue, UNITED STATES

## Abstract

**Aims:**

The aims of this study were to analyze the causes of morbidity and mortality in a large population of seabirds admitted to the Tafira Wildlife Rehabilitation Center (TWRC) in Gran Canaria Island, Spain, from 2003 to 2013, and to analyze the outcomes of the rehabilitation process.

**Methods:**

We included 1,956 seabirds (133 dead on admission and 1,823 admitted alive) in this study. Causes of morbidity were classified into nine categories: light pollution (fallout), fishing gear interaction, crude oil, poisoning/intoxication, other traumas, metabolic/nutritional disorder, orphaned young birds, other causes, and unknown/undetermined. The crude and stratified (by causes of admission) rates of the three final disposition categories (euthanasia E_r_, unassisted mortality M_r_, and release R_r_), the time until death, and the length of stay were also studied for the seabirds admitted alive.

**Results:**

Yellow-legged Gull (*Larus michahellis*) was the species most frequently admitted (46.52%), followed by Cory’s Shearwater (*Calonectris diomedea borealis*) (20.09%). The most frequent causes of morbidity were light pollution (fallout) (25.81%), poisoning/intoxication (24.69%), and other traumas (18.14%). The final disposition rates were: E_r_ = 15.35%, M_r_ = 16.29%, and R_r_ = 68.34%. The highest E_r_ was observed in the ‘other traumas’ category (58.08%). Seabirds admitted due to metabolic/nutritional disorder had the highest M_r_ (50%). The highest R_r_ was observed in the light pollution (fallout) category (99.20%).

**Conclusions:**

This survey provides useful information for the conservation of several seabird species. We suggest that at least the stratified analysis by causes of admission of the three final disposition rates, and the parameters time until death and length of stay at the center should be included in the outcome research of the rehabilitation of seabirds. The high release rate for seabirds (68.34%) achieved at the TWRC emphasizes the importance of wildlife rehabilitation centers for the conservation of seabirds.

## Introduction

Seabird population changes are good indicators of long-term and large-scale changes in marine ecosystems because their populations are strongly influenced by threats to marine and coastal ecosystems [1–3]. Global seabird population declined overall by 69.7% between 1950 and 2010, mainly due to anthropogenic causes [3]. The principal threats at sea are commercial fisheries (through overfishing of food resources and mortality on fishing gear) and pollution, whereas on land, they include alien invasive predators, habitat degradation and human disturbance [3–6]. Direct exploitation remains a problem for some species both at sea and ashore [5].

The Canary Islands harbor 11 breeding seabird species from three families [7], four of these breeding species being listed as nationally vulnerable in the National Catalogue of Threatened Species [8]. In addition, other non-breeding seabirds have also been reported in the Canary Islands [9], and some of them have an unfavorable conservation status [8,10].

Human participation through wildlife rescue networks and veterinary care in wildlife rehabilitation centers is essential for conservation purposes. The main benefits derived from the rehabilitation of wild birds are: the reinforcement of the natural population after the release, especially in endangered species, the identification of the causes of morbidity and mortality, and the regulatory changes implemented after determining human influences and causes of admission [11,12]. Data on rehabilitation of seabirds are usually focused on the causes of morbidity and mortality [13–16], but a stratified analysis by causes of the final disposition is rarely reported, being mainly focused on large oil spill events [17,18] or chronic oiling incidents [19].

The aims of this study were to analyze the causes of morbidity and mortality in a large population of seabirds admitted to the Tafira Wildlife Rehabilitation Center (TWRC) in Gran Canaria Island, Spain, from 2003 to 2013, and to analyze the outcomes of the rehabilitation process studying the crude and stratified (by causes of admission) rates of the three final disposition categories (euthanasia, unassisted mortality, and release), the time until death, and the length of stay at the rehabilitation center.

## Materials and methods

### Ethics statement

Seabird rehabilitation program at the TWRC was conducted with authorization of the Wildlife Department of the Canary Islands Government (Ms. Guacimara Medina), and the Environment Department of the Cabildo de Gran Canaria (Ms. María del Mar Arévalo). Animal work and all sampling procedures were specifically approved by the Tafira Wildlife Rehabilitation Center Animal Care Committee and the insular government Cabildo de Gran Canaria, and were consistent with standard vertebrate protocols and veterinary practices. Seabirds that had to be euthanized for animal welfare reasons were administered barbiturates by intravenous injection.

### Animals and study area

A retrospective study was performed using the original medical records of 1,956 seabirds (133 dead on admission and 1,823 alive) admitted to the TWRC, Gran Canaria Island, Spain, from 2003 to 2013. Previously, 95 seabirds admitted dead due to unknown causes had been excluded from the study. The TWRC receives seabirds mainly from Gran Canaria and sporadically from other islands of the Canary Islands archipelago. Gran Canaria (27°73’-28°18’N and 15°35’-15°83’W) is the third largest island (1,560.1 km^2^) of the Canary Islands archipelago and its coastline is 236 km long [20]. Gran Canaria Island has a permanent population of 851,150 inhabitants and the biggest mean density (546 inhabitants/km^2^) of the archipelago. In addition, Gran Canaria receives annually more than three million tourists.

### Variables analyzed

Species, gender, age, locality, date and primary cause of admission, and final disposition of each bird were recorded. Sex was determined when possible by gonadal examination at necropsy or by genetic blood analysis in some members of the Family Procellariidae. Age was categorized as “<1 year calendar” and “>1 year calendar” according to the European Union for Bird Ringing [21]. The year was divided into four seasons, spring (from March to May), summer (June to August), fall (September to November) and winter (December to February), to study the seasonality of the different causes of admission.

The primary cause of morbidity was defined as the main condition responsible for the seabird’s need for treatment. When several causes were observed in the same seabird, clinical history and complementary studies were critical to determine the primary cause of morbidity, and only this primary cause was recorded. Primary causes of morbidity were classified into nine categories: light pollution (fallout), fishing gear interaction, crude oil, poisoning/intoxication, other traumas, metabolic/nutritional disorder, orphaned young birds, other causes, and unknown/undetermined. Other traumas were subdivided into: gunshot, collision, predation, pecks (from congeners), and unknown trauma (for those cases with clinical signs of trauma but without clear evidence about the accident). The metabolic/nutritional disorder category was subdivided into: weakness, cachexia, and other diagnoses grouped by organ systems. Chicks and fledgling seabirds were included in the orphaned young category. Other causes were subdivided into: infectious/parasitic disease, birds found in water ponds, glue trap, and miscellany. The infectious/parasitic disease subcategory was applied when a pathogenic microorganism and/or parasite was confirmed by microbiological, parasitological or histopathological diagnosis. Poisoning/intoxication was diagnosed by symptoms, bioassays, and toxicological analyses.

The sources used to assign the categories mentioned above were: the physical examination carried out by the veterinarian at the time of admission; the information from the people that collected the seabird; the case history; and when available, complementary studies as radiology, hematology, blood chemistry, cytology, gross pathology, histopathology, microbiology, parasitology, and toxicology.

Three categories were established for analyzing the final disposition of the seabirds admitted alive: euthanized birds (due to poor quality of life and/or prognosis for survival in the wild), birds that died during the hospitalization period, and birds released into the wild. Therefore three percentage rates were calculated for the total of seabirds admitted alive: euthanasia rate (E_r_), unassisted mortality rate (M_r_), and release rate (R_r_). In addition, these percentage rates were also calculated for each cause of morbidity.

The parameters time until death (T_d_; number of days between the date of admission and the date of the death) for seabirds that were euthanized or died during the hospitalization period, and length of stay in the wildlife rehabilitation center for released seabirds (T_r_; number of days between the date of admission and the release date) were also analyzed for each cause of morbidity [12,20].

### Veterinary care and criteria for release

Veterinary care (stratified by causes of admission) for the seabirds admitted alive to the TWRC are summarized in S1 Table; these protocols were based on several publications reporting wildlife clinical practice guidelines, welfare rehabilitation standards, and pre-release health screening protocols [22,23]. The criteria used to determine when a bird was ready to be released included full recovery from the original injury, active behavior, self-feeding, correct weight, adequate waterproof plumage, and adequate locomotive skills [23].

### Statistical analysis

Statistical analyses were conducted using SPSS v.22.0 (SPSS Inc., Chicago IL) and R package v.3.1.0 (R Development Core Team 2014, Vienna, Austria). Chi-square test (χ^2^) or Fisher exact tests were used to determine whether there was a significant difference between proportions. Odds Ratio (OR) measure of association was employed for disease comparisons. In order to study differences among years, trend analyses were applied for specific causes with a minimum of 100 cases. Median, percentile 10 (P_10_), and percentile 90 (P_90_) for the variables T_d_ and T_r_ were calculated.

## Results

### Descriptive analyses

A total of 1,956 seabirds were included in this study, distributed in three orders: Order Procellariiformes with 915 animals of 10 different species, Order Suliformes with 53 animals of two species, and Order Charadriiformes with 988 seabirds of 9 different species. Yellow-legged Gull (*Larus michahellis*) was the species most frequently admitted (46.52%, n = 910), followed by Cory’s Shearwater (*Calonectris diomedea borealis*) (20.09%, n = 393), Bulwer’s Petrel (*Bulweria bulwerii*) (9.45%, n = 185), and White-faced Storm-Petrel (*Pelagodroma marina hypoleuca*) (8.07%, n = 158) (Table 1).

**Table 1:** Demographic data of the seabird species admitted to the Tafira Wildlife Rehabilitation Center (Gran Canaria Island, Spain) (2003–2013).

Species	Number of seabirds (%)	Sex		Age		
		M/F^a^	Unknown	<1 year	>1 year	Unknown
**Order Procellariiformes**	**915 (46.78)**	**35/55**	**825**	**207**	**317**	**391**
**Family Procellariidae**						
Cory’s Shearwater (*Calonectris diomedea borealis*)	393 (20.09)	34/54	305	38	193	162
Bulwer’s Petrel (*Bulweria bulwerii*)	185 (9.45)	0/1	184	98	33	54
Barolo Shearwater (*Puffinus baroli*)	10 (0.51)	-	10	6	1	3
Manx Shearwater (*Puffinus puffinus*)	11 (0.56)	-	11	6	3	2
Great Shearwater (*Ardenna gravis*)	7 (0.35)	1/0	6	4	1	2
Northern Fulmar (*Fulmarus glacialis*)	1 (0.05)	-	1	0	0	1
**Family Hydrobatidae**						
Leach’s Storm-Petrel (*Oceanodroma leucorhoa*)	128 (6.54)	-	128	29	17	82
Band-rumped Storm-Petrel (*Oceanodroma castro*)	16 (0.81)	-	16	4	3	9
European Storm-Petrel (*Hydrobates pelagicus pelagicus*)	6 (0.31)	-	6	1	2	3
White-faced Storm-Petrel (*Pelagodroma marina hypoleuca*)	158 (8.07)	-	158	21	64	73
**Order Suliformes**	**53 (2.71)**	**0/1**	**52**	**24**	**21**	**8**
**Family Sulidae**						
Northern Gannet (*Morus bassanus*)	52 (2.65)	0/1	51	23	21	8
**Family Phalacrocoracidae**						
European Shag (*Phalacrocorax aristotelis*)	1 (0.05)	-	1	1	0	0
**Order Charadriiformes**	**988 (50.51)**	**13/19**	**956**	**194**	**181**	**613**
**Family Laridae**						
Lesser Black-backed Gull (*Larus fuscus*)	24 (1.22)	-	24	7	11	6
Yellow-legged Gull (*Larus michahellis*)	910 (46.52)	13/18	879	183	154	573
Black-legged Kittiwake (*Rissa tridactyla*)	7 (0.35)	0/1	6	1	1	5
Herring Gull (*Larus argentatus*)	5 (0.25)	-	5	0	2	3
Black-headed Gull (*Chroicocephalus ridibundus*)	10 (0.51)	-	10	1	4	5
Common Tern (*Sterna hirundo hirundo*)	11 (0.56)	-	11	1	2	8
Sandwich Tern (*Thalasseus sandvicensis*)	19 (0.97)	-	19	1	7	11
Black Tern (*Chlidonias niger*)	1 (0.05)	-	1	0	0	1
**Family Alcidae**						
Atlantic Puffin (*Fratercula arctica*)	1 (0.05)	-	1	0	0	1
**TOTAL**	**1,956**	**48/75**	**1,833**	**425**	**519**	**1,012**

^a^M/F: male/female ratio

Gender classification resulted in 93.71% of birds (n = 1,833) classified as undetermined gender, 2.45% (n = 48) sexed as males (M), and 3.83% (n = 75) as females (F). In terms of age, 21.73% (n = 425) of all seabirds were determined to be < 1 year of age, 26.53% (n = 519) were > 1year of age, and 51.74% (n = 1,012) were of unknown age. A significantly higher number of seabirds were aged as >1 year in Cory’s Shearwater (χ^2^ = 104.0, *P* < 0.0001) and White-faced Storm-Petrel (χ^2^ = 21.73, *P* < 0.0001); conversely, Bulwer’s Petrel admissions showed a significantly higher number of birds aged as <1 year (χ^2^ = 32.25, *P* < 0.0001).

### Distribution of causes of morbidity

The number of cases and frequency distribution by causes of admission are shown in Table 2 and S2 Table. The most frequent causes of morbidity were light pollution (fallout) (25.81%, n = 505), poisoning/intoxication (24.69%, n = 483), other traumas (18.14%, n = 355), and unknown/undetermined (11.80%, n = 231). The other primary causes had frequencies below 10%. Within the group of seabirds admitted dead (n = 133), the most frequent causes of mortality were other traumas (62.40%, n = 83) and poisoning/intoxication (19.54%, n = 26). Necropsies were performed only in the cases of poisoning/intoxication (n = 26) and metabolic/nutritional disorder (n = 7).

**Table 2:** Primary causes of morbidity for 1,956 seabirds admitted to the Tafira Wildlife Rehabilitation Center (Gran Canaria Island, Spain) (2003–2013).

Cause of admission	Number of admissions			%
	Dead	Alive	Total	
**Crude oil**	5	31	36	1.8
**Fishing gear**	5	90	95	4.8
**Light pollution (fallout)**	0	505	505	25.8
**Metabolic/nutritional disorder**	7	108	115	5.9
**Orphaned young**	6	100	106	5.4
**Other causes**	1	29	30	1.5
**Other traumas**	83	272	355	18.1
Gunshot	0	1	1	0.1
Collision	2	2	4	0.2
Predation	66	8	74	3.8
Pecks	1	7	8	0.4
Unknown origin	14	254	268	13.7
**Poisoning/intoxication**	26	457	483	24.7
**Unknown/undetermined**	-	231	231	11.8

A statistical comparison of causes of admission between seabird species with more than 100 admissions is shown in S3 Table. White-faced Storm-Petrel had the highest risk of fallout (OR = 9.00; 95% CI: 6.26–12.95). Yellow-legged Gull had the highest risk of being injured by fishing gear (OR = 8.64; 95% CI: 4.68–15.95), poisoning/intoxication (OR = 36.35; 95% CI: 24.43–54.09), and unknown/undetermined causes (OR = 2.35; 95% CI: 1.76–3.13). Cory’s Shearwater showed the highest risk of other traumas (OR = 4.58; 95% CI: 3.56–5.89).

Apart from the orphaned young category (in which obviously there was a significantly higher number of birds aged < 1 year; χ^2^ = 92.16, *P* < 0.0001), two causes of admission showed significant differences when age groups were compared. A significantly higher number of birds aged >1 year was observed for crude oil (χ^2^ = 16.20, *P* < 0.0001), and other traumas (χ^2^ = 21.62, *P* < 0.0001) categories.

### Seasonality

**Fig 1:**
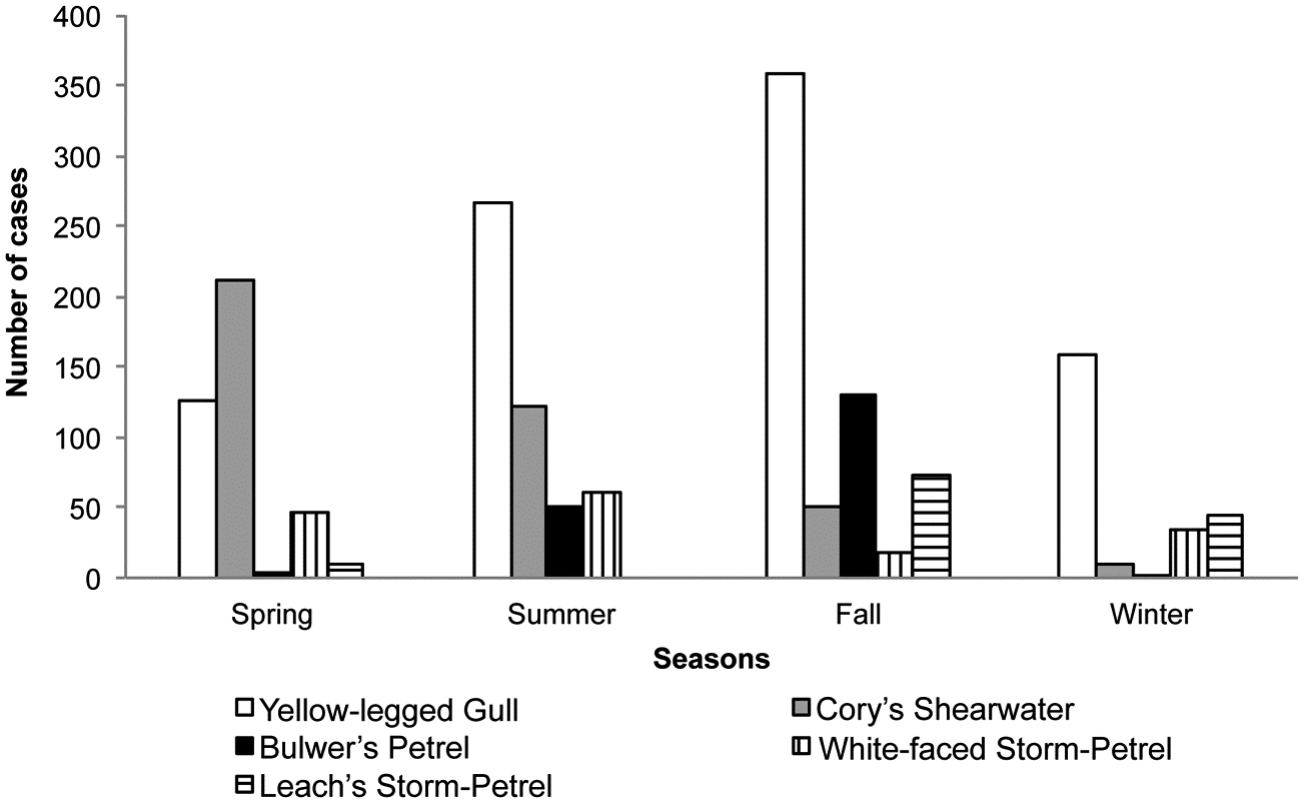
Seasonal admissions of the five most frequently admitted seabird species at the Tafira Wildlife Rehabilitation Center (2003–2013).

Admissions were distributed as follows: 36.45% (n = 713) in fall, 26.32% (n = 515) in summer, 22.13% (n = 433) in spring, and 15.08% (n = 295) in winter. Seasonal admissions of the five most frequently admitted seabird species are shown in Fig 1. Yellow-legged Gull, Bulwer’s Petrel, and Leach’s Storm-Petrel (*Oceanodroma leucorhoa*) were most frequently admitted in fall, whereas Cory’s Shearwater and White-faced Storm-Petrel were most frequently admitted in spring and summer, respectively.

**Fig 2:**
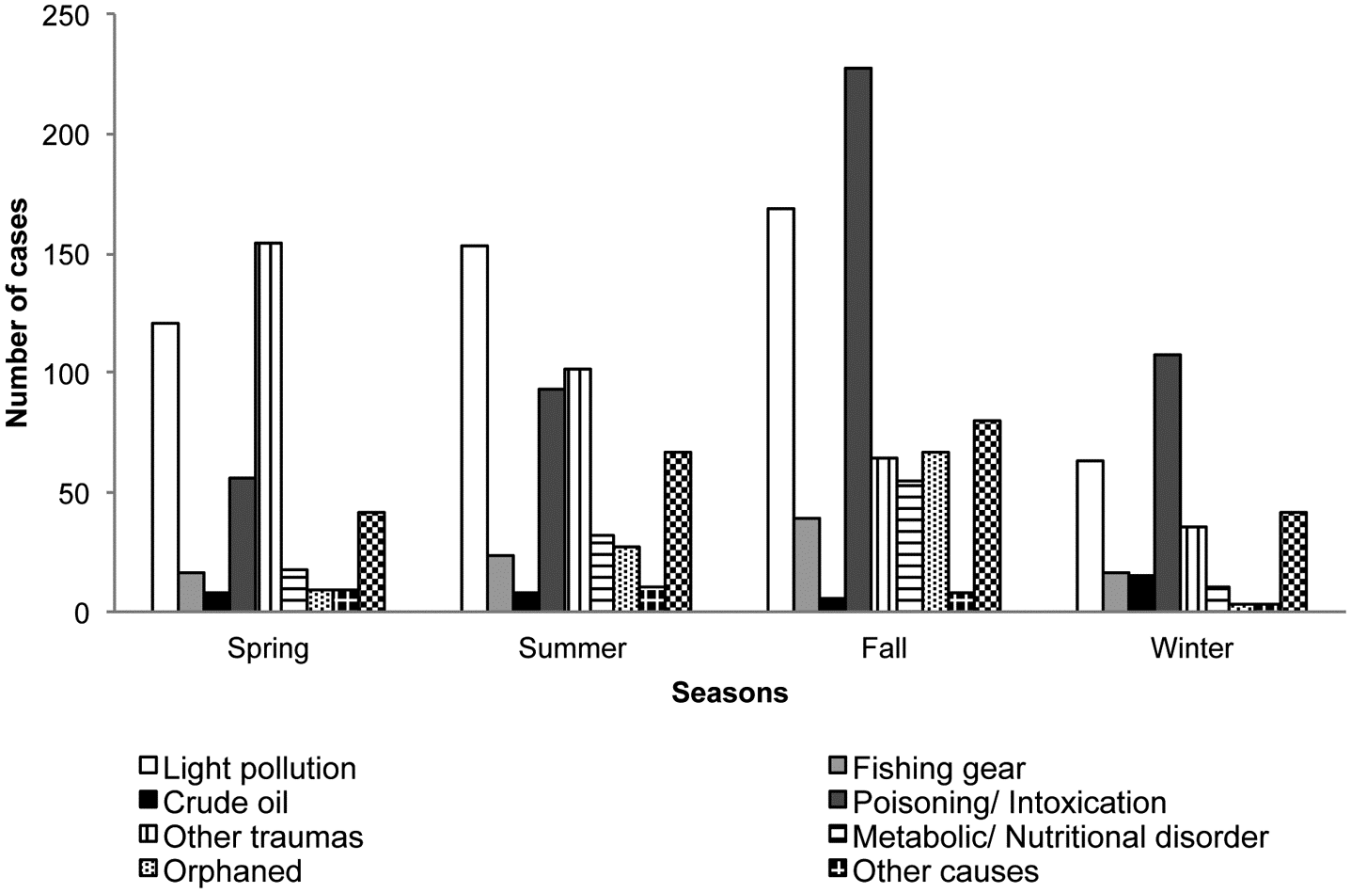
Seasonal variation in causes of admission of seabirds at the Tafira Wildlife Rehabilitation Center (2003–2013).

Seasonal variation in causes of admission is shown in Fig 2. We detected significant differences between seasons when light pollution (fallout) cases were analyzed (χ^2^ = 18.64, *P* < 0.0001), being more prevalent in fall (33.26%, n = 168) and summer (30.29%, n = 153). A significantly higher number of seabirds admitted due to fishing gear, metabolic/nutritional disorder, orphaned young birds, and unknown/undetermined causes were observed in fall and summer (χ^2^ = 14.85, *P* = 0.002; χ^2^ = 40.58, *P* < 0.0001; χ^2^ = 94.30, *P* < 0.0001; χ^2^ = 18.64, *P* < 0.0001, respectively). The highest number of other traumas was observed in spring (43.38%, n = 154) (χ^2^ = 87.91, *P* < 0.0001). Poisoning/intoxication was significantly more prevalent in fall (46.99%, n = 227) (χ^2^ = 136.15, *P* < 0.0001).

### Annual variation of admissions

**Fig 3:**
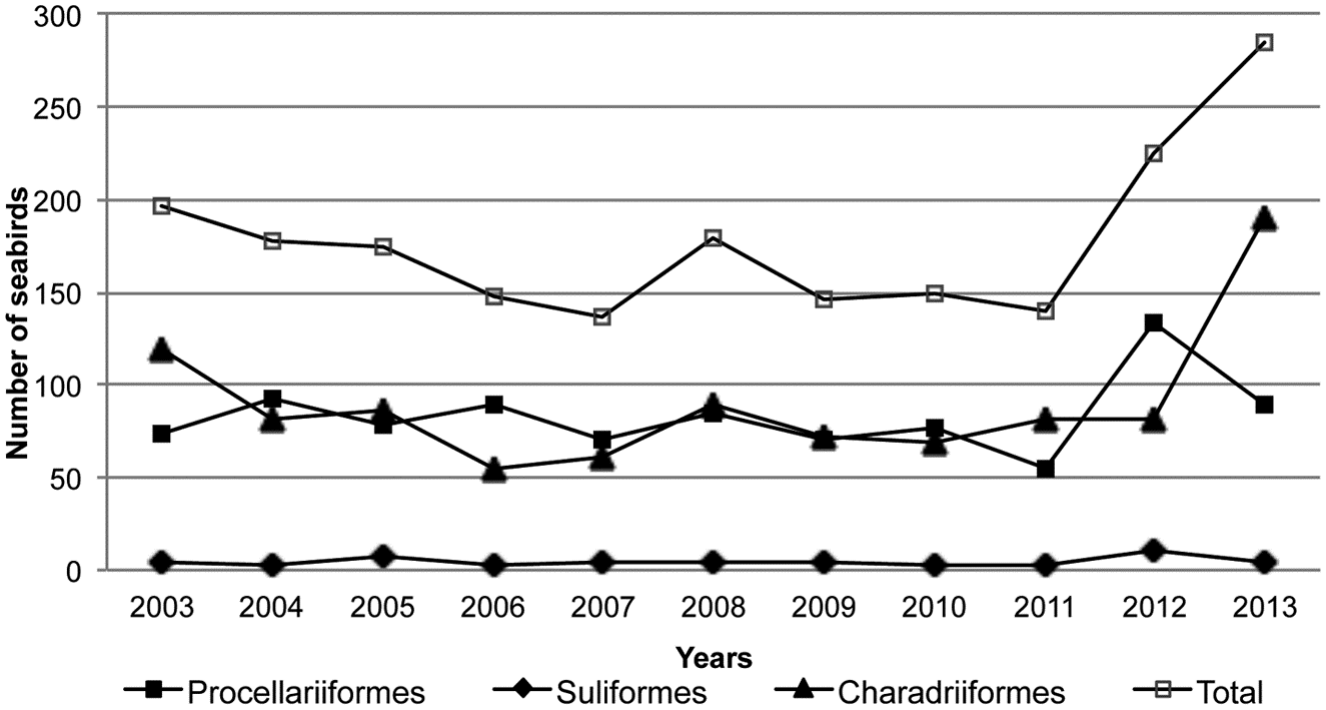
Number of seabirds admitted yearly to the Tafira Wildlife Rehabilitation Center (2003–2013) distributed by taxonomic order.

**Fig 4:**
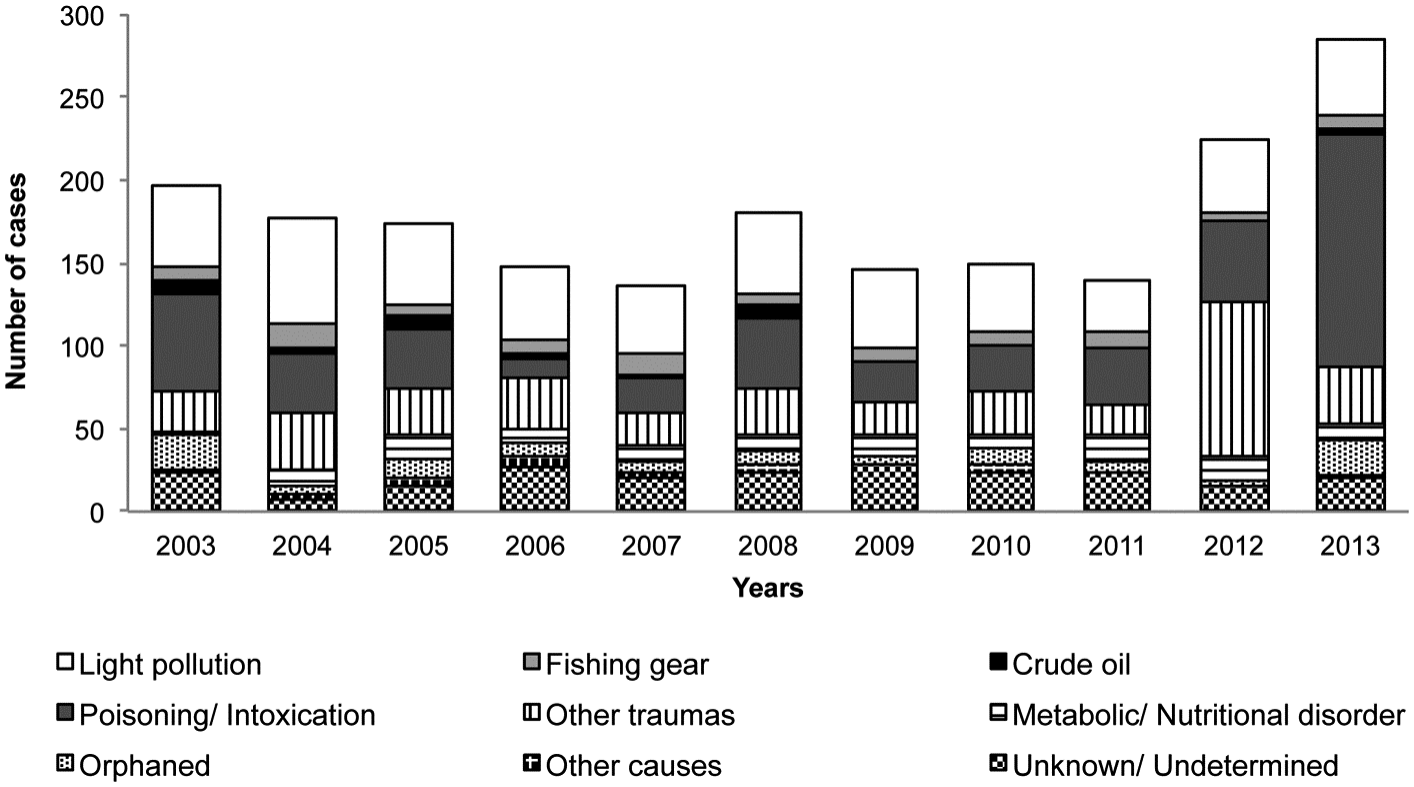
Annual variation in causes of admission of seabirds at the Tafira Wildlife Rehabilitation Center (2003–2013).

**Fig 5:**
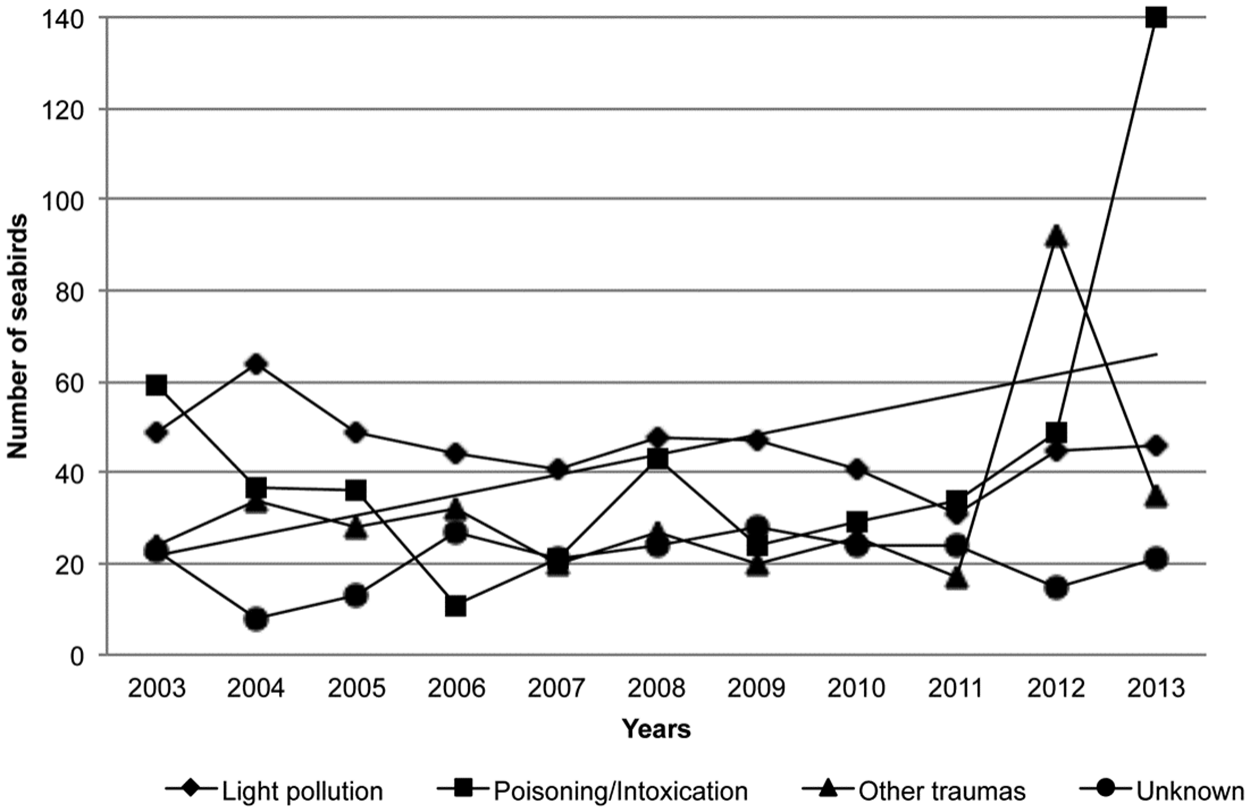
Annual variation of the four most frequent causes of seabird admissions at the Tafira Wildlife Rehabilitation Center (2003–2013).

The number of seabirds admitted yearly to the TWRC distributed by taxonomic order is shown in Fig 3. Admissions of Procellariiformes and Charadriiformes peaked in 2012 and 2013, respectively. Annual variation in causes of admission is shown in Fig 4. Although not statistically significant, the highest increase was observed in the poisoning/intoxication category (Fig 5).

### Final dispositions

The final disposition of the 1,823 seabirds admitted alive showed the following rates: E_r_ = 15.35% (n = 280), M_r_ = 16.29% (n = 297), and R_r_ = 68.34% (n = 1,246). Species from the Charadriiformes order showed a significantly higher euthanasia rate (19.21%, χ^2^ = 18.64, *P* < 0.0001) and unassisted mortality rate (23.9%, χ^2^ = 21.62, *P* < 0.0001) and a significantly lower release rate (56.88%, χ^2^ = 143.95, *P* < 0.0001) compared to the Procellariiformes order (E_r_ = 11.63%, M_r_ = 5.03%, R_r_ = 83.33%) (S4 Table).

**Fig 6:**
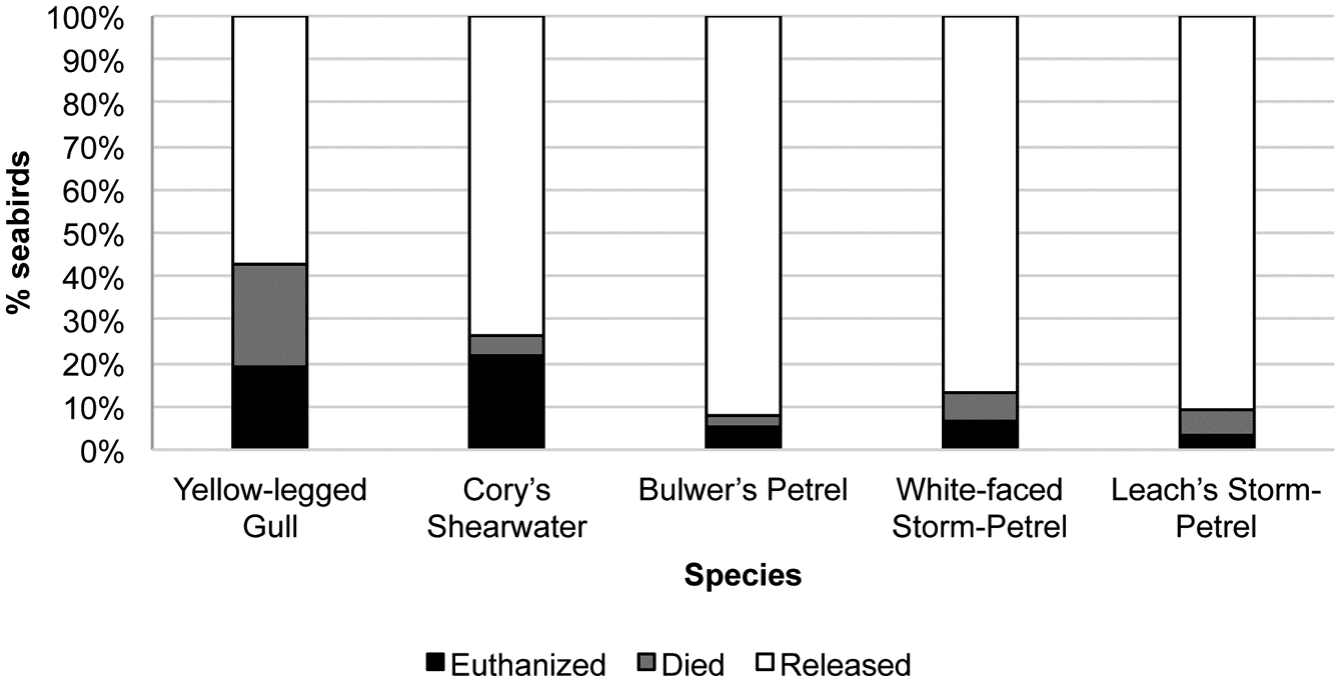
Final disposition of the five most frequently admitted seabird species at the Tafira Wildlife Rehabilitation Center (2003–2013).

The final dispositions of the five most frequently seabird species admitted (Yellow-legged Gull, Cory’s Shearwater, Bulwer’s Petrel, White-faced Storm-Petrel, and Leach’s Storm-Petrel) are shown in Fig 6. Within the Procellariiformes order, Cory’s Shearwater had a significantly higher euthanasia rate (22.01%, χ^2^ = 49.18, *P* < 0.0001), a significantly lower release rate (73.58%, χ^2^ = 33.75, *P* < 0.0001), and no significant difference in unassisted mortality rate (4.40%, χ^2^ = 0.1, *P* = 0.38) compared to the sum of Bulwer’s Petrel, White-faced Storm-Petrel, and Leach’s Storm-Petrel. Similar results were observed when Cory’s Shearwater was compared to each of these three petrel species.

The final dispositions, T_d_, and T_r_ by causes of admission are shown in Table 3. The highest E_r_ was observed in the ‘other traumas’ category (58.08%). Seabirds admitted due to metabolic/nutritional disorder and poisoning/intoxication had the highest M_r_, 50% and 27.13%, respectively. The highest R_r_ was observed in the light pollution (fallout) category (99.20%); orphaned young also showed a high R_r_ (87%).

**Table 3:** Final disposition and time of hospitalization (stratified by causes of admission) of the seabirds admitted alive to the Tafira Wildlife Rehabilitation Center (2003–2013).

Cause of admission	Number of seabirds	Final disposition											
		Euthanized				Died				Released			
		Number	E_r_ (%)	Time T_d_		Number	M_r_ (%)	Time T_d_		Number	R_r_ (%)	Time T_r_	
				Median	P_10_-P_90_			Median	P_10_-P_90_			Median	P_10_-P_90_
**Crude oil**	31	13	41.9	0	0–5.6	5	16.1	2	1–18	13	41.9	15	1.4–50.6
**Fishing gear**	90	22	24.4	0.5	0–18.6	16	17.8	2.5	0–36.4	52	57.8	6	0–17.8
**Light pollution (fallout)**	505	3	0.6	0	0–3	1	0.2	0	0–0	501	99.2	0	0–0.8
**Metabolic/nutritional disorder**	108	11	10.2	1	0–15.8	54	50	1	0–8.6	43	39.8	11	0.2–28.4
**Orphaned young**	100	4	4	0.5	0–18	9	9	3	0–26	87	87	0	0–11.2
**Other causes**	29	7	24.1	0	0–16	2	6.9	1	0–2	20	68.9	1	0–36.5
**Other traumas**	272	158	58.1	0	0–2	37	13.6	1	0–8.8	77	28.3	0	0–14.3
**Poisoning/intoxication**	457	27	5.9	2	0–16.8	124	27.1	1	0–8.6	306	66.9	12	4–31
**Unknown/undetermined**	231	35	15.1	0	0–16	49	21.2	1	0–13.8	147	63.6	2	0–16.4
**TOTAL**	**1,823**	**280**	**15.3**	**0**	**0–7**	**297**	**16.3**	**1**	**0–10**	**1,246**	**68.3**	**0**	**0–17**

T_d_: number of days between the date of admission and the date of the death.

T_r_: number of days between the date of admission and the release date.

P_10_, P_90_: percentiles 10, 90

Within the group of euthanized seabirds all categories had median T_d_ values ≤ 2 days. The median T_d_ in the seabirds that died during the hospitalization period was ≤ 3 days for all categories. Within the group of released seabirds the median time of stay in the TWRC ranged from 0 days (light pollution, other traumas, and orphaned young) to 15 days (crude oil).

## Discussion

The epidemiological studies of the causes of morbidity and mortality of birds admitted to wildlife rehabilitation centers can be used to evaluate the health status and anthropogenic threats of free-living avian species [11,24]. This study was undertaken as part of a plan to disseminate information learned during rehabilitation of wild birds at TWRC. A similar study regarding the causes of morbidity of 2,458 free-living raptors admitted to TWRC during 2003–2013 was recently published [25]; in that study, the most frequent causes of admission were trauma (33.8%), orphaned young birds (21.7%), unknown (18.4%), and metabolic/nutritional disorder (11.1%).

In the present study, 17 of the seabird species admitted at TWRC are currently included in the Spanish List of Wildlife Species with Special Protection [8]; furthermore, five of these species are also included in the Spanish Catalogue of Threatened Species as “in danger of extinction” [Black Tern (*Chlidonias niger*)], and “vulnerable” [Manx Shearwater (*Puffinus puffinus*), Band-rumped Storm-Petrel (*Oceanodroma castro*), White-faced Storm-Petrel, and European Shag (*Phalacrocorax aristotelis*)] [8].

Few studies on causes of morbidity or mortality of seabirds covered more than one decade [13,15,26–28]. While most studies only cover a short period of time, the present retrospective study included data for a long period (11 years), providing a longer term view of causes of admission as they shift over time.

Light pollution (fallout) was the most frequent cause of admission (25.81%), mainly among Procellariiformes species. For these seabirds, which commonly attend breeding colonies at night, light pollution has been reported to cause disorientation [4,29–33] and increased predation rates by gulls [34]. In a 9-year study on light-induced morbidity and mortality among Procellariiformes on Tenerife Island, the majority of the petrels that were found grounded were Cory’s Shearwaters (93.4%) [4]; in addition, authors estimated that between 45 and 61% of fledglings were affected by artificial light attraction. In another survey on Tenerife Island, it was demonstrated that late Cory’s Shearwater fledglings stranded by lights showing abundant down were more susceptible to fatal collisions and that the lights did not selectively kill birds with lower body condition indices [35]. More recently, the use of GPS data-loggers demonstrated that birds were grounded at locations closer than 16 km from colonies on their maiden flights, and 50% were rescued within a 3 km radius from the nest-site [36]. In our study, the three species most frequently admitted due to light pollution were Cory’s Shearwater (34.06%), White-faced Storm-Petrel (22.37%), and Bulwer’s Petrel (21.78%). Several hypotheses have been proposed to explain this attraction to artificial lights: physiology and morphology of their retinas [37], need for visual cues for navigation [4,38], and inexperience, particularly in adults of non-breeding species [4]. Because light pollution admissions are concentrated around the fledgling periods of the different species, an intensive light reduction during these periods or some changes in light signature have been proposed [4]. Use of shielding lights preventing upward radiation was proved to be very effective in Hawaii [39].

Poisoning/intoxication represented the second major cause of admission (24.69%), and 94.20% of these cases were diagnosed in Yellow-legged gulls. Botulism was diagnosed in the majority of cases and was probably associated with opportunistic feeding at the landfill; in fact, some earthworks at the landfill exposed previous waste and caused an increase in the number of admissions in 2013. Several outbreaks of botulism have been reported affecting several seabird species in different locations around the world [27,40,41]. Botulism occurs following the ingestion of various neurotoxins produced by the bacterium *Clostridium botulinum* [40]. Poisoning due to rodenticides and other unidentified toxic substances were involved in the remaining cases, probably also associated with feeding at the municipal landfill. No investigations were conducted to rule out the participation of harmful dinoflagellate toxins; however, harmful algal toxins have been suggested to have caused or contributed to mortality events in seabirds [14,42,43].

The ‘other traumas’ category was the third cause of admission (18.14%); the significantly higher number of birds aged >1 year in this category may be due to the fact that these birds are more resistant to these injuries and therefore have a better chance of being found alive and transferred to the rehabilitation center; in addition, adult birds are more numerous and therefore are more available to be injured. Within this admission category, trauma of unknown origin was the most frequent cause of admission (13.70% of the total). The unknown origin of the traumas makes it difficult to suggest specific preventive measures. Predation was the second cause of other traumas (3.78% of the total). Predation from invasive alien species, such as rats and cats, has been identified as an important threat to survival of seabirds [3,5]. In our study, 89.19% of the predation cases were reported in a single attack of feral dogs on a colony of Cory’s shearwaters. Despite efforts by local and island authorities through awareness campaigns, there is a need for legal actions to increase the severity of punishments for people who abandon dogs.

Weakness, cachexia, and other diagnoses grouped by organ systems were included in the metabolic/nutritional disorder category (5.87%). Emaciation was associated with several Great Shearwater (*Ardenna gravis*) mortality events along the Eastern coast of the United States [15] and stranded seabirds along the German North Sea coast [13]. Within the group of seabirds admitted due to other causes, the prevalence of infectious/parasitic disease was very low in our study (0.15%). This prevalence was lower than those reported in other surveys [13,27]. However, the impact of underlying infectious or parasitic diseases was underestimated because complete microbiological and parasitological analyses were not done routinely in all cases due to financial constraints. And probably in our study, many of these cases contributed to increase the metabolic/nutritional disorder category.

Fishing gear interaction has been identified as an important threat for seabirds [3,5,44]. In our study, the percentage of seabirds admitted due to fishing gear (4.85%) was lower than expected, especially considering the prevalence of interaction with fishing gear in other vertebrate species, such as sea turtles, reported in the Canary Islands [20,45]. Probably the higher mortality rates of seabirds at sea compared to sea turtles made the number of birds sent to the TWRC to be much lower.

Admissions due to crude oil were low (1.84%). In a previous study on crude oil as stranding cause among loggerhead sea turtles (*Caretta caretta*) in the Canary Islands, authors concluded that the designation of the Canary Islands as a Particularly Sensitive Sea Area (PSSA) in 2005 by the International Maritime Organization (IMO) was associated with positive effects on the reduction of sea turtle strandings caused by crude oil [46]. Similarly as suggested before for ‘other traumas’ category, the significantly higher number of birds aged >1 year in the crude oil category may be due to a higher tolerance and therefore a better chance of being found alive and sent to the rehabilitation facility; in addition, adult birds may be more numerous than young-of-the-year and therefore more of them are available to become oiled. Age groups may also have different foraging areas that put them at different risks of contamination; perhaps fledglings have not yet learned bad habits like hanging around harbors.

Some seasonal variations (species admissions and causes of admission) were clearly related to the specific breeding seasons. Fledging periods of the breeding seabird species with more than 100 admissions range from summer (White-faced Storm-Petrel, Yellow-legged Gull) to fall (Cory’s Shearwater, Bulwer’s Petrel) [9]. Bulwer’s Petrels were most frequently admitted in fall because 79.46% of admissions of this species occurred due to light pollution and orphaned young. These two causes were also involved in 73.42% of admissions of White-faced Storm-Petrels, so they were most frequently admitted in summer. However, in the case of Cory’s Shearwaters, less than 50% of admissions were attributed to these two causes, so they were not most frequently admitted in fall, but in spring.

As mentioned before, the increasing tendency observed in the number of seabirds admitted due to intoxication was associated in most cases to inappropriate management of waste at the landfill. In the absence of an incineration plant in Gran Canaria Island, the destination of most of the waste is landfilling, but landfills occupy large spaces and there are difficulties in finding new locations in a territory particularly limited by insularity.

Our retrospective study revealed the need to improve several diagnostic protocols at the TWRC, mainly necropsy and diagnosis of parasitic and infectious diseases. Post-mortem examination of all birds admitted dead or deceased while under care is essential to determine the cause of death and identify potential outbreaks as early as possible [47]. In addition, a complete necropsy would have made it possible to identify the sex of these birds, thus improving the dataset. As mentioned before, the impact of infectious/parasitic diseases was underestimated because complete microbiological and parasitological analyses were not done routinely in all cases due to financial limitations. However, data from rehabilitation centers can provide valuable insight on the occurrence of infectious agents within wild populations [48], and the pathology of parasites that are otherwise uncommon or from hosts that are infrequently sampled in the wild [49].

In our study, 68.34% of seabirds admitted alive to the TWRC were successfully released, and 31.64% of seabird admissions resulted in euthanasia or unassisted mortality. References on the final dispositions of seabird rehabilitation are scarce because most surveys usually have been focused on the causes of mortality [13,15,24,26,27]. In addition, a comparative analysis between studies of the final dispositions of seabirds in wildlife centers is difficult due to the heterogeneity of the surveys [14,17,18].

Euthanasia, accordingly to animal welfare protocols, is a final option in all wildlife species rehabilitation [50]. Within the primary causes of morbidity with more than 100 admissions, the euthanasia rate was notably higher in the ‘other traumas’ category (58.08%) compared to other admission categories. The severity of their lesions explains the generally poor prognosis for these birds.

Unassisted mortality rate has been used as a quality indicator parameter in rehabilitation of raptors [12] and, recently, in rehabilitation of sea turtles [20]. In our study, within the primary causes of morbidity with more than 100 admissions, the unassisted mortality rate was notably higher in the metabolic/nutritional disorder (50%) and poisoning/intoxication (27.13%) categories compared to other causes of admission. Several factors, including poor condition on arrival to the TWRC due to time taken for capture, insufficient body reserves to deal with the stress of rehabilitation and handling, the need to develop techniques to improve care of these poor condition seabirds, and underlying inaccurately diagnosed conditions in the metabolic/nutritional disorder category, could explain these results.

In our study, the highest release rate was achieved in the light pollution (fallout) category (99.20%); this value was similar to that previously reported on Tenerife Island (94.7%) [4], but those authors doubted about the survival rate of the released birds. The high release rate observed in the orphaned young category (87%) was due to the fact that the majority of birds within this category were older fledglings with limited ability to fly, but healthy at the time of admission.

For rehabilitation to be classed as successful the bird must be re-established back into the wild population and have a similar chance to those of wild birds of entering the breeding pool [51]. Several studies suggested poor post-release survival of seabirds when considering individual species like Common Murre (*Uria aalge*), Northern Gannet (*Morus bassanus*), and Common Scoter (*Melanitta nigra*) [51,52]. However, when radio-telemetry was used in a post-release survival study on Common Murre following the 1999 *Stuyvesant* oil spill, authors found that the difference in survival between rehabilitated and control birds mostly occurred during the first 34 days after release; after that period of time, they survived comparably to non-oiled control murres [53]. In a more recent study on African Penguin (*Spheniscus demersus*) chicks abandoned by moulting parents, post-release survival rates were similar to African Penguin chicks reared after oil spills and to survival rates for naturally-reared birds [54].

In the present survey, all the statistically significant differences detected in the final disposition rates when compared among seabird species were clearly related to the cause of admission; this suggests that the different causes of admission with their different prognoses determine the final disposition rates.

The parameter time to death, previously used in rehabilitation of raptors, provides direct insight into the initial assessment and prognostication, the complete rehabilitation process, and the validity of veterinary protocols [12]. In our study, all categories had median T_d_ values ≤ 2 days, meaning that the decision was made very soon based on the poor prognosis of these cases. The median T_d_ in the seabirds that died during the hospitalization period was ≤ 3 days for all categories, suggesting that these first days of stay at the facility are critical, and a complete clinical evaluation should be performed on these birds, despite their apparently less severe presentation.

Several authors recommended that stay at wildlife rehabilitation centers must be as short as possible to reduce the risk of captive-related complications, infectious diseases, and behavioral disorders [12,55]. According to our results, seabirds admitted due to contamination by crude oil represent an especially important consumer of time and efforts.

In conclusion, this survey is the first large-scale study on causes of morbidity and mortality of seabirds on Gran Canaria Island, providing useful information for the conservation of these birds. We suggest that, as proposed previously for the rehabilitation of raptors [12], at least the stratified analysis by causes of admission of the three final disposition rates (E_r_, M_r_, and R_r_), and the parameters time until death (T_d_) and length of stay at the center (T_r_) should be included in the outcome research of the rehabilitation of seabirds. Finally, although the high release rate for seabirds (68.34%) achieved at the TWRC emphasizes the importance of wildlife rehabilitation centers for the conservation of seabirds, further studies would be necessary to know the post-release survival rate of rehabilitated seabirds and to understand the effects of rehabilitation on the conservation of seabird populations in Gran Canaria Island.

## Supporting information

S1 Table Veterinary care for the seabird species admitted alive to the Tafira Wildlife Rehabilitation Center (2003–2013). (PDF)

S2 Table Primary causes of morbidity for 1,956 seabirds admitted to the Tafira Wildlife Rehabilitation Center (Gran Canaria Island, Spain) (2003–2013). (PDF)

S3 Table Causes of admission of seabirds to the TWRC Center (2003–2013) and statistical comparison between species with more than 100 admissions. (PDF)

S4 Table Final disposition of the seabird species admitted alive to the Tafira Wildlife Rehabilitation Center (2003–2013). (PDF)

S1 File Data of seabirds admitted at the TWRC during the period 2003–2013. (XLSX)
