# Supplementary material for: A long-term retrospective study on rehabilitation of seabirds in Gran Canaria Island, Spain (2003-2013)
Source: PLoS One. 2017 May 5;12(5):e0177366. doi: 10.1371/journal.pone.0177366 (PMC5419649; doi:10.1371/journal.pone.0177366)
Supplement: S1 Table — (PDF) [file pone.0177366.s001.pdf]

S1 Table. Veterinary care for the seabird species admitted alive to the Tafira Wildlife Rehabilitation Center (2003-2013).

Cause of admission	Care protocol
**Crude oil**	Cleaning of mouth, nares and cloaca with physiological saline<br>Eye cleaning (when necessary)<br>Hydrocarbon removal using a rag or towel; cleaning with hot water and degreaser soap; rinsing and drying<br>Oral fluid therapy<br>Appropriate and palatable nutrition
**Fishing gear**	Fish hook surgical removal<br>Wound cleaning and disinfection<br>Anti-inflammatory therapy<br>Antibiotic therapy<br>Oral fluid therapy<br>Appropriate and palatable nutrition
**Light pollution (fallout)**	Oral fluid therapy (when necessary)
**Metabolic/nutritional disorder**	Oral fluid therapy<br>Appropriate and palatable nutrition (if necessary, tube feeding)
**Orphaned young**	Appropriate and palatable nutrition
**Other causes**	Drying (when found in water ponds)<br>Glue removal using petroleum; cleaning with hot water and degreaser soap; rinsing and drying<br>Oral fluid therapy<br>Appropriate and palatable nutrition (if necessary, tube feeding)
**Other traumas**	Surgical removal (gun pellets)<br>Wound cleaning and disinfection<br>Anti-inflammatory therapy<br>Antibiotic therapy<br>Oral fluid therapy<br>Appropriate and palatable nutrition (if necessary, tube feeding)
**Poisoning/intoxication**	Oral fluid therapy<br>Appropriate and palatable nutrition (if necessary, tube feeding)
**Unknown/undetermined**	Oral fluid therapy<br>Appropriate and palatable nutrition (if necessary, tube feeding)<br>Anti-inflammatory therapy (when necessary)<br>Antibiotic therapy (when necessary)
