# Supplementary material for: A long-term retrospective study on rehabilitation of seabirds in Gran Canaria Island, Spain (2003-2013)
Source: PLoS One. 2017 May 5;12(5):e0177366. doi: 10.1371/journal.pone.0177366 (PMC5419649; doi:10.1371/journal.pone.0177366)
Supplement: S2 Table — (PDF) [file pone.0177366.s002.pdf]

S2 Table. Primary causes of morbidity for 1,956 seabirds admitted to the Tafira Wildlife Rehabilitation Center (Gran Canaria Island, Spain) (2003-2013).

**CAUSE OF ADMISSION**	**ORDER PROCELLARIIFORMES**										**ORDER SULIFORMES**	
	Calonectris diomedea borealis	*Bulweria bulwerii*	*Puffinus baroli*	*Puffinus puffinus*	*Ardenna gravis*	*Fulmarus glacialis*	*Oceanodroma leucorhoa*	*Oceanodroma castro*	*Hydrobates pelagicus pelagicus*	*Pelagodroma marina hypoleuca*	*Morus bassanus*	*Phalacrocorax aristotelis*
**Crude oil**	3	1	0	0	0	0	4	0	1	3	6	0
**Fishing gear**	4	0	0	0	0	0	0	0	0	0	4	0
**Light pollution (fallout)**	172	110	7	7	4	0	75	13	4	113	0	0
**Metabolic/nutritional disorder:**	5	2	1	0	0	0	4	2	0	6	30	0
Weakness	3	1	1	0	0	0	4	1	0	4	19	0
Cachexia	1	0	0	0	0	0	0	0	0	1	8	0
Others[a]	1	1	0	0	0	0	0	1	0	1	3	0
**Orphaned young:**	21	37	1	0	2	0	6	0	0	3	2	0
Chicks	4	0	0	0	0	0	0	0	0	0	0	0
Fledglings	17	37	1	0	2	0	6	0	0	3	2	0
**Other causes:**	6	3	0	0	0	0	2	0	0	3	3	0
Infectious/parasitic disease	0	0	0	0	0	0	0	0	0	0	0	0
Water pond	4	1	0	0	0	0	1	0	0	1	2	0
Glue trap	1	2	0	0	0	0	0	0	0	0	0	0
Miscellany	1	0	0	0	0	0	1	0	0	2	1	0
**Other traumas:**	157	23	1	2	1	0	15	1	1	22	3	0
Gunshot	0	0	0	0	0	0	0	0	0	0	0	0
Collision	2	0	0	0	0	0	0	0	0	1	0	0
Predation	71	1	0	0	0	0	1	0	0	1	0	0
Peck	0	0	0	0	0	0	0	0	0	0	0	0
Unknown origin	84	22	1	2	1	0	14	1	1	20	3	0
**Poisoning/ intoxication**	0	0	0	0	0	0	0	0	0	0	0	0
**Unknown/ undetermined**	25	9	0	2	0	1	22	0	0	8	4	1
**TOTAL no. seabirds**	**393**	**185**	**10**	**11**	**7**	**1**	**128**	**16**	**6**	**158**	**52**	**1**
**% seabirds**	**20.09**	**9.45**	**0.51**	**0.56**	**0.35**	**0.05**	**6.54**	**0.81**	**0.3**	**8.07**	**2.65**	**0.05**

Table S2 (continued). Primary causes of morbidity for 1,956 seabirds admitted to the Tafira Wildlife Rehabilitation Center (Gran Canaria Island, Spain) (2003-2013).

CAUSE OF ADMISSION	ORDER CHARADRIIFORMES									TOTAL no. cases	%
	Larus fuscus	*Larus michahellis*	*Rissa tridactyla*	*Larus argentatus*	*Chroicocephalus ridibundus*	*Sterna hirundo hirundo*	*Thalasseus sandvicensis*	*Chlidonias niger*	*Fratercula arctica*		
**Crude oil**	0	9	1	0	1	2	5	0	0	**36**	**1.84**
**Fishing gear**	1	83	1	0	0	1	1	0	0	**95**	**4.85**
**Light pollution (fallout)**	0	0	0	0	0	0	0	0	0	**505**	**25.81**
**Metabolic/nutritional disorder:**	0	56	3	0	1	2	2	1	0	**115**	**5.87**
Weakness	0	30	1	0	1	1	1	1	0	**68**	**3.47**
Cachexia	0	15	1	0	0	1	1	0	0	**28**	**1.43**
Others[a]	0	11	1	0	0	0	0	0	0	**19**	**0.97**
**Orphaned young:**	1	31	0	0	0	1	1	0	0	**106**	**5.41**
Chicks	0	1	0	0	0	0	0	0	0	**5**	**0.25**
Fledglings	1	30	0	0	0	1	1	0	0	**101**	**5.16**
**Other causes:**	0	12	0	0	0	0	1	0	0	**30**	**1.53**
Infectious/parasitic disease	0	3	0	0	0	0	0	0	0	**3**	**0.15**
Water pond	0	1	0	0	0	0	1	0	0	**11**	**0.56**
Glue trap	0	0	0	0	0	0	0	0	0	**3**	**0.15**
Miscellany	0	8	0	0	0	0	0	0	0	**13**	**0.66**
**Other traumas:**	1	114	0	2	1	3	7	0	1	**355**	**18.14**
Gunshot	0	0	0	0	0	1	0	0	0	**1**	**0.05**
Collision	0	1	0	0	0	0	0	0	0	**4**	**0.2**
Predation	0	0	0	0	0	0	0	0	0	**74**	**3.78**
Peck	0	8	0	0	0	0	0	0	0	**8**	**0.4**
Unknown origin	1	105	0	2	1	2	7	0	1	**268**	**13.7**
**Poisoning/ intoxication**	18	455	2	2	6	0	0	0	0	**483**	**24.69**
**Unknown/ undetermined**	3	150	0	1	1	2	2	0	0	**231**	**11.8**
**TOTAL no. seabirds**	**24**	**910**	**7**	**5**	**10**	**11**	**19**	**1**	**1**	**1,956**	
**% seabirds**	**1.22**	**46.52**	**0.35**	**0.25**	**0.51**	**0.56**	**0.97**	**0.05**	**0.05**		

[a] Other systemic diseases: respiratory, digestive, etc.
