# Supplementary material for: A long-term retrospective study on rehabilitation of seabirds in Gran Canaria Island, Spain (2003-2013)
Source: PLoS One. 2017 May 5;12(5):e0177366. doi: 10.1371/journal.pone.0177366 (PMC5419649; doi:10.1371/journal.pone.0177366)
Supplement: S3 Table — (PDF) [file pone.0177366.s003.pdf]

S3 Table. Causes of admission of seabirds to the TWRC Center (2003-2013) and statistical comparison between species with more than 100 admissions.

CAUSE OF ADMISSION	Number	%	OR (CI 95%) [*P*-value]				
			Calonectris diomedea borealis	***Bulweria bulwerii***	***Oceanodroma leucorhoa***	***Pelagodroma marina hypoleuca***	***Larus michahellis***
**Crude oil**	36	1.84	0.35 (0.1-1.16) [ns]	0.27 (0.03-1.97) [ns]	1.81 (0.63-5.2) [ns]	1.03 (0.31-3.41) [ns]	0.37 (0.17-0.8) [0.009]
**Fishing gear**	95	4.85	0.16 (0.06-0.45) [<0.0001]	0	0	0	8.64 (4.68-15.95) [<0.0001]
**Light pollution (fallout)**	505	25.81	2.87 (2.27-3.63) [<0.0001]	5.1 (3.73-6.99) [<0.0001]	4.6 (3.18-6.64) [<0.0001]	9 (6.26-12.95) [<0.0001]	0
**Metabolic/nutritional disorder**	115	5.87	0.17 (0.07-0.42) [<0.0001]	0.16 (0.04-0.65) [0.004]	0.49 (0.18-1.37) [ns]	0.61 (0.26-1.41) [ns]	1.09 (0.75-1.59) [ns]
Weakness	68	3.47	0.08 (0.02-0.28) [<0.0001]	0.11 (0.01-0.81) [0.009]	1.39 (0.46-4.17) [ns]	1.05 (0.36-3.1) [ns]	1.53 (0.91-2.55) [ns]
Cachexia	28	1.43	0.07 (0.01-0.57) [0.001]	0	0	0.61 (0.08-4.64) [ns]	2.21 (1.03-4.74) [0.03]
Others[a]	19	0.97	0.11 (0.01-0.89) [0.013]	0.45 (0.06-345) [ns]	0	0.93 (0.12-7.2) [ns]	2.62 (1.03-6.61) [0.035]
**Orphaned Young:**	106	5.41	0.98 (0.6-1.6) [ns]	6.16 (3.99-9.51) [<0.0001]	0.85 (0.36-1.97) [ns]	0.31 (0.1-1.01) [0.041]	0.45 (0.29-0.7) [<0.0001]
Chicks	5	0.25	8.99 (0.99-81.02) [0.018]	0	0	0	0.45 (0.51-4.13) [ns]
Fledglings	101	5.16	0.39 (0.22-0.68) [0.001]	9.84 (5.64-17.17) [<0.0001]	1.45 (0.57-3.7) [ns]	0.46 (0.14-1.56) [ns]	0.74 (0.46-1.18) [ns]
**Other causes:**	30	1.53	0.99 (0.4-2.44) [ns]	1.06 (0.32-3.54) [ns]	1.02 (0.24-4.33) [ns]	1.27 (0.38-4.23) [ns]	0.76 (0.36-1.59) [ns]
Infectious/parasitic disease	3	0.15	0	0	0	0	0
Water pond	11	0.56	1.26 (0.36-4.37) [ns]	0.83 (0.1-6.58) [ns]	2.18 (0.27-17.74) [ns]	1.7 (0.21-13.7) [ns]	0.18 (0.02-1.42) [ns]
Glue trap	3	0.15	1.1 (0.09-12.24) [ns]	17.14 (1.53-191.74) [0.002]	0	0	0
Miscellany	13	0.66	0.18 (0.02-1.39) [ns]	0	1.81 (0.22-14.51) [ns]	3.18 (0.67-14.99) [ns]	3.02 (0.97-9.37) [0.044]
**Other traumas**	355	18.14	4.58 (3.56-5.89) [<0.0001]	0.61 (0.39-0.96) [0.03]	0.58 (0.33-1.01) [ns]	0.71 (0.44-1.13) [ns]	0.47 (0.37-0.61) [<0.0001]
Gunshot	1	0.05	0	0	0	0	0
Collision	4	0.2	2.21 (0.31-15.87) [ns]	0	0	5.74 (0.58-56.76) [ns]	0.61 (0.06-5.93) [ns]
Predation	74	3.78	83.03 (25.68-268.39) [<0.0001]	0.1 (0.01-0.73) [0.005]	0.26 (0.03-1.99) [ns]	0.2 (0.02-1.53) [ns]	0
Peck	8	0.4	0	0	0	0	0
Unknown origin	268	13.7	1.01 (0.71-1.43) [ns]	0.61 (0.35-1.05) [ns]	1.37 (0.63-2.98) [ns]	1.86 (0.92-3.76) [ns]	1.37 (0.98-1.91) [ns]
**Poisoning/intoxication**	483	24.69	0	0	0	0	36.35 (24.43-54.09) [<0.0001]
**Unknown/ undetermined**	231	11.8	0.44 (0.29-0.68) [<0.0001]	0.35 (0.18-0.7) [0.002]	1.6 (0.99-2.6) [ns]	0.37 (0.18-0.77) [0.006]	2.35 (1.76-3.13) [<0.0001]

ns: no statistical significance (P>0.05); [a] Other systemic diseases: respiratory, digestive, etc.
