# Supplementary material for: A long-term retrospective study on rehabilitation of seabirds in Gran Canaria Island, Spain (2003-2013)
Source: PLoS One. 2017 May 5;12(5):e0177366. doi: 10.1371/journal.pone.0177366 (PMC5419649; doi:10.1371/journal.pone.0177366)
Supplement: S4 Table — (PDF) [file pone.0177366.s004.pdf]

S4 Table. Final disposition of the seabird species admitted alive to the Tafira Wildlife Rehabilitation Center (2003-2013).

**Species**	**Number of seabirds**	**Final disposition**					
		**Euthanized**		**Died**		**Released**	
		**Number**	**E_r (%)**	**Number**	**M_r (%)**	**Number**	**R_r (%)**
**Order Procellariiformes**	834	97	11.63	42	5.03	695	83.33
**Family Procellariidae**							
Cory's Shearwater (*Calonectris diomedea borealis*)	318	70	22.01	14	4.4	234	73.58
Bulwer's Petrel (*Bulweria bulwerii*)	182	10	5.49	5	2.74	167	91.75
Barolo Shearwater (*Puffinus baroli*)	10	0	0	1	10	9	90
Manx Shearwater (*Puffinus puffinus*)	11	0	0	2	18.18	9	81.81
Great Shearwater (*Ardenna gravis*)	7	1	14.28	0	0	6	85.71
Northern Fulmar (*Fulmarus glacialis*)	1	0	0	0	0	1	100
**Family Hydrobatidae**							
Leach's Storm-Petrel (*Oceanodroma leucorhoa*)	126	4	3.17	8	6.34	114	90.47
Band-rumped Storm-Petrel (*Oceanodroma castro*)	16	1	6.25	1	6.25	14	87.5
European Storm-Petrel (*Hydrobates pelagicus pelagicus*)	6	1	16.67	0	0	5	83.33
White-faced Storm-Petrel (*Pelagodroma marina hypoleuca*)	157	10	6.36	11	7.01	136	86.62
**Order Suliformes**	52	3	5.76	31	59.61	18	34.61
**Family Sulidae**							
Northern Gannet (*Morus bassanus*)	51	3	5.88	31	60.78	17	33.33
**Family Phalacrocoracidae**							
European Shag (*Phalacrocorax aristotelis*)	1	0	0	0	0	1	100
**Order Charadriiformes**	937	180	19.21	224	23.9	533	56.88
**Family Laridae**							
Lesser Black-backed Gull (*Larus fuscus*)	24	4	16.67	5	20.83	15	62.5
Yellow-legged Gull (*Larus michahellis*)	863	166	19.23	202	23.4	495	57.35
Black-legged Kittiwake (*Rissa tridactyla*)	6	1	16.67	3	50	2	33.33
Herring Gull (*Larus argentatus*)	5	1	20	1	20	3	60
Black-headed Gull (*Chroicocephalus ridibundus*)	9	0	0	3	33.33	6	66.67
Common Tern (*Sterna hirundo hirundo*)	10	2	20	6	60	2	20
Sandwich Tern (*Thalasseus sandvicensis*)	18	5	27.78	4	22.22	9	50
Black Tern (*Chlidonias niger*)	1	0	0	0	0	1	100
**Family Alcidae**							
Atlantic Puffin (*Fratercula arctica*)	1	1	100	0	0	0	0
**TOTAL**	**1,823**	**280**	**15.35**	**297**	**16.29**	**1,246**	**68.34**
